# Supplementary material for: Fish harvesting advice under climate change: A risk-equivalent empirical approach
Source: PLoS One. 2021 Feb 19;16(2):e0239503. doi: 10.1371/journal.pone.0239503 (PMC7895391; doi:10.1371/journal.pone.0239503)
Supplement: S1 Appendix — (PDF) [file pone.0239503.s001.pdf]

Duplisea, Roux, Hunter, Rice. Risk-based fishery harvesting advice under climate change: a risk-equivalent empirical approach

Appendix 1: *derivation of surplus production and how it can be used as the basis of an empirical model of stock growth influenced a climate variable or any other variable that affects stock production*

Surplus production has a long history of use as a means of determining population growth and influence of external factors on that growth (Russel 1931, Dickie et al. 1972, Banse and Mosher 1980, Kell et al. 2012). Although it is has been described, we rederive the model to show how we used it as an empirical modelling approach for looking at impacts of climate change on sustainable fisheries strategies.

When we refer to stock productivity, we mean the addition or loss of biomass of a stock over time, in this case over a year. The main productivity processes of an exploited fish stock are: recruitment (R_t), individual growth (G_t), natural mortality (M_t) and fishery removals (C_t). Because we are assuming a bounded stock, we do not consider immigration or emigration of biomass from the stock area. Total production from one year to the next is therefore the sum total of the production processes and because R_t and G_t are always positive and M_t and C_t are always negative natural mortality and fishery removals are always negative we can write the production (P_t) equation as:

Eq. 1 $$P_t = R_t + G_t - M_t - C_t$$

Given a time series of measures representing stock biomass we can difference the estimates between years which gives us a measure of P_t:

Eq. 2 $$P_t = B_{t+1} - B_t$$

Which we term ΔB which makes it clear that annual net production can simply be estimated as the difference in an observable time biomass series

Eq. 3 $$\Delta B_t = P_t$$

We also not that we usually have a time series of annual fisheries removals (C_t) from a stock. Therefore, we can combine eq. 3 with eq. 1 and move our two measureables (ΔB_t, C_t) to one side of the equation putting the less measureables (R_t, G_t, M_t) on the other side we get

Eq. 4 $$\Delta B_t + C_t = R_t + G_t - M_t$$

We now get an equation that separates measurable from unmeasurable processes (note that they are unmeasurable in the sense that they are not readily available in a data moderate situation and their estimation may require elaborate experiments or sophisticated statistical modelling approaches). We are most interested in the right side of this equation because these are the natural components of production and through our data (biomass time series and catch time series) we can have a measure of their sum total. We call this natural production ${}^{n}P_t$ with the n superscript to reflect that it is the natural productivity components

Eq. 5 $${}^{n}P_t = R_t + G_t - M_t$$

We also know that ${}^{n}P_t$ is the component of stock production which is affected by CC or any other external variable that affects stock production. We also know that these rates are a function of the biomass present at a time B_t, i.e. so the recruitment and growth and mortality in biomass terms is a function of how much biomass is there. So tidying-up eq. 4 we can see that the natural production processs on the right hand side can be estimated in sum total by adding catch to the difference in biomass from one year to the next and we can also divide this quantity (Eq. 5) by biomass to get a specific rate of natural production and bring it back to measureable terms (Eq. 4)

Eq. 6 $$\frac{{}^{n}P_t}{B_t} = \frac{\Delta B_t + C_t}{B_t}$$

and as described above we know that this specific rate of natural stock production is a function of the externally forced productivity conditions of the stock with some error

Eq. 7 $$\frac{{}^{n}P_t}{B_t} = f(E_t) + \varepsilon_t$$

Where E_t is an external variable and ε_t is an additive error term.

Fishery catch can simply be modelled as the exploitation rate multiplied by the biomass

Eq. 8 $$C_t = B_t \cdot F_t$$

Where F_t is the fishery exploitation rate in year *t*.

We can now write an equation for the model showing how future biomass can be determined from past biomass combined with a specific rate of production removing commercial fish catch

Eq. 9 $$B_{t+1} = B_t + B_t \cdot \frac{{}^{n}P_t}{B_t} - C_t$$

A density dependent extension of equation 9 can be written employing a sigmoidal function (as in the Schaefer model) where the growth rate of the population approaches 0 as the biomass of the population approaches its carrying capacity (*K*).

Eq. 10 $$B_{t+1} = B_t + B_t \cdot \frac{{}^{n}P_t}{B_t} \cdot \left[1 - \frac{B_t}{K}\right]^{\theta} - C_t$$

Where ϑ is a term describing the asymmetry in the density dependence and when ϑ =0, there is no density dependence.

Thus equation 10 describes the basic model which is the same as the Schaeffer surplus production model. In practical application here through this model is not fitted to data but annual growth rates (surplus production) are calculated directly from the data and given a relationship (fitted or assumed) between an E variable and those growth rates and a distribution for plausible future values of and E variable, a rate is calculated from a sampled E and inserted into the model to create thousands of stochastic projections.

The density dependent version of the model (eq 10) was only used for sensitivity purposes where a K=3*max(Bt) was assumed and ϑ=0.5 (Schaefer model). In reality, for stocks that have been fished for some time, they are usually well below their carrying capacity (usually 50-66% below for optimally managed stocks) and the density dependent term has very little impact on the model outcomes. Therefore in practice the model where $\vartheta=0$, is the main model with fewer assumptions.
