# Supplementary material for: Fish harvesting advice under climate change: A risk-equivalent empirical approach
Source: PLoS One. 2021 Feb 19;16(2):e0239503. doi: 10.1371/journal.pone.0239503 (PMC7895391; doi:10.1371/journal.pone.0239503)
Supplement: S2 Appendix — (HTML) [file pone.0239503.s002.html]

Appendix A: Choosing E variables


# Appendix A: Choosing E variables

#### *Daniel Duplisea, Marie-Julie Roux, Karen Hunter, Jake Rice*

Summer 2019

## What is the best E variable?

For the Gulf of St Lawrence turbot there are 38 environmental
variables representing temperature, salinity and oxygen
concentrations in different places at different depths. It is
not always clear what is the most appropriate variable or
combination of variables to use. The purpose here is to
condition fisheries advice for turbot to plausible climate
changes that will occur over the useful time span of the
advice. So if the goal is to see how make a stock recover to a
target biomass value in 10 years, then the projections should
be done at the time span of 10 years and the climate variable
should is hypothesised to have these impacts in that 10 year
period.

For the turbot example here, temperature and oxygen levels
tend to relate closely to each other and it is usually assumed
that temperature strongly determines oxygen levels while both
clearly can have hypothesised impacts on the productivity of
turbot. The first step is therefore to try to determine the
most relevant variable that relates to turbot production,
concentrating on temperature.

Our approach to this has been to fit parametric (polynomials)
and non-paramertric (GAM) relating the P/B ratio to the
environmental variables and extract the AIC values from each
model fit. The analysis should not be determined by AIC value
alone however as the shpe of the P/B vs E relationship should
also make sense from a mechanstic point of view. That is, we
approached the analysis assuming that some kind of optimum
relationship relationship exist between turbot production and
the environmental variable. We acknowledge, however, that the
available data may not fully explore the useful range for all
variables so we may only have observed part of that
relationship. So for example an optimum relationship is
conceivably dome- shaped but we only observe a portion of that
dome in which can it may look like a linear decreasing
relationship. What we are unlikley to observe, however, is an
increasing level of production at an extreme of the observed
environmental variable. We therefore do not seriously consider
those kinds of relationship plausible and if they are observed
it is mos likely because the environmental variable is aliased
with a more important environmental driver of production.

```
row.names(gamaic)=names(tmp)[-1]
gamaic[,-1]
```

```
##                        aic.gam aic.gam.ad aic.poly aic.cubic
## Year                  47.17241   47.17241 49.14062  50.78451
## E                     48.12580   46.27304 49.31864  51.19128
## Gulf.T150             49.16692   48.53460 50.14982  51.88028
## Gulf.T200             49.37678   49.35498 49.72273  51.61557
## Gulf.T250             49.82518   49.80925 50.35199  52.29680
## Gulf.T300             49.67327   49.67327 51.05765  52.98340
## Estuary.T150          47.83435   46.75407 49.19780  50.42205
## Estuary.T200          47.57056   47.57056 49.35073  50.82269
## Estuary.T250          47.13828   47.13828 49.13638  50.80141
## Estuary.T300          40.74715   40.17561 47.42672  43.70320
## NWGulf.T150           48.99561   48.20313 50.91699  52.86797
## NWGulf.T200           48.21598   48.21598 50.05666  52.01349
## NWGulf.T250           48.40049   48.40049 50.33732  51.29042
## NWGulf.T300           47.50035   47.50035 49.09355  50.38445
## AnticostiChannel.T150 50.13543   50.13543 51.90705  53.88748
## AnticostiChannel.T200 50.14933   50.14933 51.93195  53.89183
## AnticostiChannel.T250 50.05430   50.05430 51.86127  53.49685
## MecatinaTrough.T150   41.99486   41.99486 43.55089  45.18502
## MecatinaTrough.T200   41.74707   41.74707 43.74604  45.72406
## EsquimanChannel.T150  49.38142   49.06470 50.06990  51.41116
## EsquimanChannel.T200  49.01836   48.97523 49.56468  51.10847
## EsquimanChannel.T250  50.10790   48.91986 51.63534  53.33358
## CentralGulf.T150      48.12580   46.27304 49.31864  51.19128
## CentralGulf.T200      49.81940   49.68457 50.11950  52.08584
## CentralGulf.T250      49.85607   43.24906 50.17109  51.73778
## CentralGulf.T300      50.04111   50.04111 51.34207  53.34153
## CabotStrait.T150      48.77986   48.63876 48.51959  50.51173
## CabotStrait.T200      48.73780   48.49026 48.67980  50.10419
## CabotStrait.T250      49.14214   48.54417 49.48876  51.33745
## CabotStrait.T300      50.10039   49.30276 50.93988  52.93734
## O2.mMol               50.14615   50.14615 51.81865  53.78971
## O2.saturation         50.07183   50.07183 51.76794  53.76661
```

```
mn= min(apply(gamaic[-1,-1],2,min))
mn
```

```
## [1] 40.17561
```

## Acknowledgements

Thanks for Peter Galbraith for the various temperature
variables, Denis Gilbert provided the Oxygen concentration
data for the estuary. Johanne Gauthier provided all the turbot
survey and catch data.

## References

Galbraith, P.S. 2006. Winter water masses in the Gulf of
St. Lawrence, Journal of Geophysical Research,
111:C06022. doi:10.1029/2005JC003159.

Peklova, I., Hussey, N. E., Hedges, K. J., Treble, M. A.,
& Fisk, A. T. 2012. Depth and temperature preferences of
the deepwater flatfish Greenland halibut Reinhardtius
hippoglossoides in an Arctic marine ecosystem. Marine Ecology
Progress Series, 467, 193-205.
